# *Rickettsia* Species: Genetic Variability, Vectors, and Rickettsiosis—A Review

**DOI:** 10.3390/pathogens13080661

**Published:** 2024-08-06

**Authors:** Anna Rymaszewska, Mariusz Piotrowski

**Affiliations:** 1 Department of Genetics and Genomics, Institute of Biology, University of Szczecin, ul. Felczaka 3C, 71-412 Szczecin, Poland; mariusz.piotrowski@bio-space.pl; 2 BIOSPACE Foundation, ul. Karpia 31, 61-619 Poznań, Poland

**Keywords:** tick-borne diseases, genetic diversity, rickettsiae, vectors

## Abstract

Rickettsiae are an interesting group of bacteria comprising a large number of obligate intracellular species. The circulation of these bacteria in the environment depends on the presence of vectors (blood-sucking invertebrates) and their hosts. On the basis of phylogenetic analyses in 2022, a division into five groups of *Rickettsia* has been proposed: I belli group, II canadensis group, III typhus group, and IV and V spotted group fever (respectively II, phylogenetically older, and I). The genus *Rickettsia* includes species that are both pathogenic and nonpathogenic to humans and domestic and wild animals. Some *Rickettsia* species are invertebrate symbionts. Currently, rickettsiae, which are transmitted mainly by ticks, are spreading worldwide. This has been promoted by climate change, environmental changes caused by humans, and the synanthropisation of plants and animals. Therefore, it is extremely important to monitor the natural and urban environments. The study of potential vectors and reservoirs of bacteria in the genus *Rickettsia* should be a permanent part of the analysis of the modern human environment.

## 1. Introduction

Rickettsia, which comprises a large number of species, is an extremely interesting group of bacteria. Many of these bacteria are pathogenic to humans and domestic animals. All of these are obligated intracellularly. The circulation of rickettsiae in the environment depends on the presence of vectors and hosts. Vectors include blood-sucking invertebrates, mainly ticks, lice, and fleas, which become infected during blood feeding [1]. Spread in the environment is facilitated by vertebrates that act as reservoir hosts for bacteria, such as birds, rodents, and ungulates. As reservoir hosts for rickettsiae, vertebrates often carry them over long distances, facilitating the spread of bacteria into new environments. Ticks also act as reservoir hosts, and the occurrence of rickettsiae coincides with the geographic spread of ticks. Rickettsiae are a highly diverse, broad group of bacteria that infect their invertebrate hosts and can coexist in symbiosis or as parasites or pathogens [2].

In recent years, the number of rickettsiosis cases and cases of *Rickettsia* species transmission has increased significantly. This is primarily due to new diagnostic methods based on achievements in molecular biology. New diagnostic approaches are related to next generation sequencing (NGS) and nanopore sequencing (NS) technology. The advantage of these methods over the classical Sanger sequencing is that metabarcoding can be performed. The initial material is environmental DNA (eDNA), and the analysis shows simultaneous amplicons of several target markers from multiple samples [3]. According to Stewart and Stewart [4], modern sequencing techniques have revealed a greater number of *Rickettsia* spp. infections; hence, these methods are desirable for diagnostics. However, thus far, they have been limited to a few references and research laboratories [4]. The purpose of this article is to present the most important information related to the pathogens of the *Rickettsia* genus, their classification, and their circulation in the environment.

## 2. Genetic Basis of Bacterial Classification

**Figure 1:**
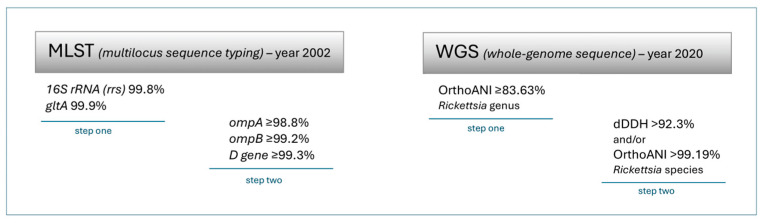
The scheme for classification of rickettsial isolates. The percentage value corresponds to similarity within the genus or species. Detailed explanations in text (Section 2).

Numerous species have been isolated within the genus *Rickettsia* and new species are still being discovered. There are reports in the literature on rickettsiae which are classified as new species, such as *Candidatus* (well-characterized but as-yet uncultured organisms), *Candidatus* R. rioja, *Candidatus* R. kotlanii, and *Candidatus* R. moreli [5,6]. It is not possible to classify bacteria into a species based on a biological criterion (ability to crossbreed and give fertile offspring); therefore, the possibility of using DNA is the best solution. Thus, the *report of the* ad hoc *committee for the re-evaluation of the species definition in bacteriology* [7] was issued in 2002, which indicated the necessity of sequencing at least five genes, including 16S rRNA and protein-coding genes (genes encoding metabolic functions and housekeeping genes). Multilocus sequence typing (MLST) using housekeeping genes has been indicated as a tool for the effective classification of bacteria. Fournier et al. [8], based on the analyses of panbacterial genes, have found a similarity within particular groups of *Rickettsia* at the genus level: ≥98.1% similarity for *rrs* (16S rRNA encoding) and 86.5% for *gltA* (citrate synthase encoding). Therefore, the taxonomic ordering of bacteria obtained from natural sources should be performed according to strictly defined rules. According to the authors, the molecular classification of rickettsiae using analyses of DNA isolates should be based on the 16S rRNA and *gltA* genes in the first place. The similarity of an isolate’s nucleotide sequence to any approved species of *Rickettsia* should be at least 99.8% for *rrs* and 99.9% for *gltA*. It is also recommended that analyses of genes encoding the high-molecular-weight peripheral proteins, *ompA* (rOmpA encoding), *ompB* (rOmpB encoding), and the *D* gene (PS120 encoding) be performed (Figure 1). The similarity of an isolated nucleotide sequence to any species of *Rickettsia* should be ≥98.8, 99.2, and 99.3%, respectively [8].

In 2020, Diop et al. [9] used a whole-genome sequence (WGS) analysis to assess several taxonomic parameters. Based on this, they proposed guidelines for a new classification of rickettsial isolates. This new classification was based on the definition of threshold values for OrthoANI (average nucleotide identity by orthology) and dDDH (digital DDH = digital DNA-DNA hybridisation dDDh). The proposed analysis consisted of two steps: classification at the genus level: OrthoANI ≥ 83.63% and at the species level: dDDH > 92.3% and/or OrthoANI > 99.19 (Figure 1). The authors showed a high correlation between the classic classification method based on MLST and the use of tools based on genome analysis. At the same time, they indicated the methods as more sensitive and accurate for distinguishing rickettsiae [9].

## 3. Rickettsia Genomes and Division into Bacterial Groups

Historically, the term ‘rickettsiae’ referred to small, intracellular bacteria. In the 1990s, the molecular analysis of the 16S rRNA gene (*rrs*) introduced significant changes to the classification of this group of bacteria. Initially, the division of the genus *Rickettsia* into groups was based on phenotypic characteristics, mainly ecological and epidemiological characteristics [9].

Contemporary molecular tools provide researchers with numerous possibilities. Detailed genomic studies of bacteria belonging to *Rickettsia* species have provided new insights into the evolutionary history of these bacteria and the resulting regularities. Gillespie et al. [10] proposed a modern version of the *Rickettsia* classification and division into four groups: the ancestral group (AG), typhus group (TG), transitional group (TRG), and spotted fever group (SFG) rickettsiae. The ancestral group includes non-pathogenic endosymbiotic species with the longest history of *R. bellii* and *R. canadensis*. The second group (TG) comprises *R. prowazekii* and *R. typhi*, which cause typhus. The TGR group includes *R. felis* (murine typhus-like) and *R. akari* (rickettsial pox). The last group of bacteria causing spotted fevers is the most abundant. It comprises bacteria with different levels of pathogenicity [10,11], and new species are being described, the pathogenicity of which has not always been confirmed [5,6,12]. In 2022, a more detailed division of *Rickettsia* was proposed [13,14]. The analysis of whole bacterial genomes collected from the databases enabled the introduction of changes to the above-mentioned classification. Based on phylogenetic analyses, the *Rickettsia* species were divided into five groups: I belli group (the oldest, initial group for *Rickettsia*); BG, II canadensis group; CG, III typhus group; TG, IV spotted fever group II (earlier TGR); and V spotted fever group I (Table 1). Group SFG II was enlarged to include a few species, such as *R. hoogstraalii*, which has a large genome in the *Rickettsia* species, that is, 2.3 Mb. El Karkouri et al. [13] also reported the presence of 41 plasmids in rickettsiae, wherein 11 of them were new and described for the first time. More than half of the *Rickettsia* species have at least one plasmid. Most species and/or strains of *Rickettsia* with the discovered plasmids are representative of SFG I and SFG II. In contrast, no plasmids were detected in the genomes of rickettsiae from group III-TG. However, genome analysis showed the presence of pseudogenes with high homology to rickettsial plasmid genes [8].

Extremely interesting are the latest analyses of the genomes of *Rickettsia* classified *R. helvetica* as an independent clade. Phylogenetic connections indicate that these bacteria are an initial group for TG, but not for SFG, as previously believed [13]. Detailed studies of the *R. helvetica* genome showed the presence of some non-synonymous substitutions as well as indel mutations with regard to representatives of the groups SFGII (TGR), TG, CG, and BG.

A similar situation occurs in *R. canadensis* (group II—CG), which currently represents a separate clade in the phylogenetic analysis [13,14]. Previously, these bacteria were grouped together with the endosymbiotic bacterium *R. bellii* and constituted an ancestral group. Shi et al. [14] suggested that *R. canadensis* bacteria were pathogenic, and they lost their pathogenicity as a result of evolution. First discovered in Ontario, Canada, in 1967 by McKeil et al. [12,14] in ticks collected from rabbits, they were soon described in many other countries, including Europe and Asia. Extensive studies on the pathogenicity of *R. canadensis* conducted in the 1970s could suggest that these bacteria might cause disease in humans. The patients had antibodies against *R. canadensis*, but it was not certain if this was caused by a cross-reaction. No confirmed cases of this disease have been identified [9]. Such phenomena were observed earlier in *R. peacocki* [14].

*R. bellii* (group I—BG) was described in 1983 in the USA by Philip et al. [15]. It showed intermediate features between rickettsiae from the group of spotted fevers and typhus. A detailed genome analysis identified it as the initial species for both groups [16]. The basis for the phylogenetic analyses was the 23S rRNA gene, as it is more informative than 16S rRNA. Stothard et al. [13] showed that the genus *Rickettsia* is a distinct, ancient lineage of the α subgroup of the *Proteobacteria.* The North American continent, where *R. bellii* is the most widespread, has been indicated as a source of origin. Studies conducted by Krawczak et al. [17] did not clearly point to North America or South America as the primary sources of origin of *R. bellii.* According to the researchers, the North American strains of these bacteria were isolated only from *Dermacentor* ticks, whereas the South American strains were isolated from *Amblyomma*, *Ixodes*, and *Haemaphysalis.*

In 2023, interesting molecular research was published on the species identified as *Candidatus* Rickettsia mendelii, discovered in the Czech Republic in 2016 [18]. The analysis of the 16S rRNA and *gltA* gene sequences indicated that this bacterium is similar to *R. canadensis* and *R. bellii*. However, there was insufficient information to determine the taxonomic status of the bacterium. Multilocus genotyping of five genetic fragments (16S rRNA, *gltA*, *ompB* genes, *groESL* operon, and the 23S–5S IGS region) allowed the rickettsiae to be determined. Igolkina et al. [19] showed that the *Ca.* R. mendelii forms a distinct phylogenetic group, which like the *R. bellii* group is a basal one.

## 4. Bacteria of the Genus *Rickettsia* in the Environment

### 4.1. Ticks as Vectors of Pathogens

Currently, more than 890 tick species have been described, including approximately 702 hard ticks (ixodidae) and 193 soft ticks (argasidae). Hard ticks include, for example, species of the genera *Amblyomma*, *Dermacentor*, *Haemaphysalis*, *Hyalomma*, *Ixodes*, and *Rhipicephalus* [6,20,21,22]. Ticks are the most important vectors for pathogens worldwide. After mosquitoes, they rank second as the main vectors of infectious diseases transmitted to humans [23].

Tick–host–pathogen interactions are significant from the point of view of medical and veterinary studies. They may shed light on the spread of infectious agents in the environment, thereby preventing tick-borne diseases. Therefore, environmental monitoring was conducted, which involved the assessment of the degree of tick infection with pathogens. Field studies should be supported by laboratory testing. Thus, hypotheses made during studies in the natural environment can be verified [24].

Tick infection is estimated by detecting the genetic material of microorganisms potentially occurring in ticks in a given area. However, the detection of DNA in a tick does not allow researchers to call it a vector. Kahl et al. [25] proposed conditions predisposing an ectoparasite to the role of a transmitter of infectious microorganisms, i.e., (i) feeding on the blood of infected vertebrates; (ii) becoming infected with a pathogen during feeding; (iii) maintaining the pathogen for at least one transformation stage; and (iv) transmitting the pathogen to another host during feeding in the next stage. The presence of the genetic material of infectious agents in ticks or vertebrates gives them the status of a host. Likewise, the detection of antibodies in the host serum indicates only the contact of the animal with an infectious agent [24]. The effectiveness with which the host transmits pathogens to the ticks feeding thereon is called infectivity. Vector capacity indicates the potential of a tick to transmit the pathogen. Vector competence defines the tick’s usefulness as a vector. This depends on numerous variables and is sensitive to environmental and biotic factors [24]. The determination of these factors enables the epidemiological characterization of the examined environment.

### 4.2. Rickettsiae as Symbionts

The common ancestor of Rickettsiales was presumably free-living. The transition to an intracellular lifestyle probably occurred 775–525 million years ago [5]. An analysis of the evolutionary history of these bacteria showed that members of the *Rickettsia* clade originated as symbionts of microeukaryotes. Over time, the bacteria of the *Rickettsia* genus evolved into arthropod symbionts. Some members of this group have become pathogenic in vertebrate animals [2].

Among invertebrates, rickettsiae have a wide range of vectors. There seems to be a certain degree of regularity. Pathogenic *Rickettsia* species have a wider spectrum of invertebrate hosts, including several genera or orders (for example, *R. aeschlimanii* were detected in ticks of the genus *Hylomma*, *Rhipicephalus* or *Heamaphysalis* [26], *R. felis* in cat and dog flea, *Ctenocephalides felis*, *C. canis*, hedgehog flea *Archeopsylla erinacei*, human flea *Pulex irritans*, rat flea *Xenopsylla cheopis*, and rodent flea *Anomiopsyllus nudata*) [27,28]. One of the earliest described species of the genus *Rickettsia*, *R. prowazeki*, is transmitted by human lice *Pediculus humans*, squirrel flea *Orchopeas howardi*, and ticks *Amblyomma cajennense*, while *R. typhi* is transmitted by rat flea *X. cheopis*, cat flea *C. felis*, and rodent lice *Leptopsylla segnis*. The ancestral species, *R. bellii* or *R. canadensis*, which are not pathogenic or have unconfirmed virulence, are vectored by hard ticks, such as *Dermacentor*, *Ixodes*, and *Amblyomma*. Thus, it may be concluded that the ability of TG and SFG rickettsiae to use other invertebrates as “hosts” is a secondary feature acquired during the course of evolution [16]. Nonpathogenic rickettsiae may be of high importance in the circulation of pathogenic species and strains. Their presence in egg cells of the vector-host in the form of a symbiotic relationship limits the possibility of secondary infection with pathogenic species, while simultaneously limiting transovarial transmission [29]. Macaluso et al. [29] have demonstrated by experiment that *R. rhipicephali* and *R. montana* (currently *R. montanensis*) are mutually excluded in the females of *Dermacentor variabilis.* Steiner et al. [30] showed that the occurrence of rickettsial endosymbionts in *I. scapularis* males reduces the rate of infection with *B. burgdorferi.*

For some invertebrate species, the rickettsiae *Rickettsia* are symbionts. The coexistence of animals with bacteria has been described in annelids *Torix tagoi* and *Hemicrepsis marginata*, multiple species of ixodid (hard ticks) and argasid (soft ticks) ticks, and beetle arthropods [17,31,32]. Symbiotic coexistence with females of selected invertebrate species may lead to the manipulation of their reproduction and to the occurrence of cytoplasmic incompatibility between females and males. This phenomenon was first described in 1994 in the ladybird beetle *Adalia bipunctata*, where the presence of the rickettsiae *Rickettsia* in female cells led to the death of male embryos [33]. Similar relationships have been detected in a few other beetle species. Speciation may be a remote consequence of manipulating the reproduction of invertebrates and symbiotic hosts. The bacteria of the *Rickettsia* genus may also be responsible for the parthenogenetic reproduction of some insect species, such as booklouse *Liposcelis bostrychophila*, *Neochrysocharis formosa*, and *sweet potato* whitefly *Bemisia tabaci* [32,34].

Ticks, as vectors of *Rickettsia* species, are extremely important for them. Ticks have limited mobility and are typically transmitted over short distances. However, as shown by Kagemann and Clay [35], some pathogens can affect tick motility. Thus, rickettsiae increased and the bacterium *Arsenophonus* decreased the locomotion abilities of the larvae of three tick species from North America: *Am. americanum*, *D. variabilis*, and *I. scapularis* [35].

Vertebrates are most often involved in the spread of ticks. These ectoparasites that feed on the blood of animals, for example, small rodents, deer, or birds, are transferred to new environments. Moreover, each developmental stage must take blood to undergo a transformation, and feeding itself is a slow and lengthy process. This results in the potential possibility of tick infection, thereby spreading the bacteria in the environment and increasing its extent of occurrence. An important contributing factor is anthropopression. As a result of human activity, the environment has been undergoing significant changes in recent years. Alien plant species penetrate ecosystems of rural as well as urban areas. Synanthropic plants spread in the environment, changing the species composition of native vegetation. These changes can impact the local fauna, including the spread of ticks, potential vectors of pathogens.

### 4.3. The Lifecycle and Spread of Rickettsia

The life cycle of a tick involves stages of larvae, nymphs, and adults (imago). The condition of transformation into the next stage is a blood meal from the host, which supplies the necessary energy. At that time, ticks may acquire viruses, bacteria, or protozoa. Thus, they are introduced into the epidemiological chain of a given pathogen [24]. Tick host transmission is defined as a horizontal transfer.

Once the acquired pathogen persists in the tick population by transstadial transmission (TS), that is, during molting, the pathogens remain in an already infected individual. In the case of some pathogens, we may encounter another vertical transmission of pathogens, called transgenerational transmission. The most common is the transovarial transmission (TO) of an infected female onto eggs. Transspermal transmission (sperm of infected males) has been observed only in a single species of tick and selected species of *Rickettsia* [12,24,36,37]. Effective transovarial transmission makes the larval stage a threat to the potential hosts of ticks. Adult males are characterised by short or no feeding times [37,38,39]. However, nymphs and females are the greatest threats [24]. Ticks may also become infected during co-feeding [24,37].

A very interesting study on the transmission of pathogens was conducted by Buczek et al. [40]. In sympatric ticks, they observed numerous contacts between *I. ricinus* males and *D. reticulatus* females [40,41]. The authors suggested that the oral–anal contact between these tick species was probably stimulated by *B. burgdorferi* and/or *Rickettsia* spp. [40].

Some researchers have indicated a certain regularity in the circulation of bacteria in the tick population. SFG *Rickettsia* spp. with low virulence were maintained in the tick population via vertical transmission. By contrast, virulent SFG species prefer horizontal transmission. In this way, they protect ticks against the negative effects of infection, such as the restriction of vitality and reproduction [42]. Throughout evolution, ticks have developed various ways of feeding, typical in a given species. Specific stages may feed on various individuals through each transformation outside the host. Most ticks experience this kind of three-host cycle, that is, each active stage falls on the plant litter after feeding, where molting occurs. Some tick species have developed life cycles related to these two hosts. In this case, larva–nymph transformation occurs in the host [24]. There are no rules regarding the selection of hosts by ticks. Some tick species are highly specific with regard to feeding, whereas others are not. Host specificity in ticks results from various factors, including existence in various biogeographic areas and phylogenetic and physiological conditions. The hosts of *Ixodes* spp. are primarily birds (especially Passeriformes), rodents, wild ruminants, and carnivores [43,44]. In most cases, humans act as accidental hosts [24].

An interesting fact from an epidemiological perspective is that the transmission of pathogens to the host does not occur directly after a tick bite. It usually takes at least a few hours or even days to affect the transmission of pathogenic agents [45,46]. The call signal for microorganisms may be the temperature of the vertebrate host or a portion of the blood collected by the tick [47]. The delayed transmission of pathogens to the host is necessary for the prevention of tick-borne diseases. New products which block the transmission of tick-borne pathogens have been tested and marketed [48,49,50]. However, this is a significant challenge for chemical and pharmaceutical companies. Experiments have shown that individual compounds are not effective in fighting ectoparasites. The best results can be achieved using a combination of various chemical compounds. One must also consider the potential harm to the host and environment [47].

## 5. Rickettsiosis

More than 90% of tick-borne bacteria belong to two orders, Spirochaetales and Rickettsiales [34]. Lyme borreliosis is the most common disease caused by spirochaetes of the genus *Borrelia* [34,48]. Various *Borrelia* species cause different pathological conditions ranging from mild to severe, which persist for many years [37,51]. Bacteria of the order Rickettsiales cause rickettsioses, anaplasmoses or ehrlichioses [51].

Rickettsioses are commonly defined in the medical environment as typhus. However, this is an oversimplification. Numerous pathogenic species of rickettsiae and diseases resulting in different signs and symptoms, course, and mortality have been described. Rickettsioses are characterised by symptoms typical of tick-borne diseases such as fever, headache, and muscle pain. Common signs of rickettsioses are rashes of various specificities, such as maculopapular rash, macular rash, or petechial rash. A characteristic feature of infection by various species of rickettsiae is the crust. The site of the tick bite appears to be a crusty necrotic lesion with or without a surrounding erythematous halo [1,52].

Rocky Mountain Spotted Fever (RMSF) was one of the first rickettsioses described, in which *R. rickettsii* was found to be the etiological factor. It was first diagnosed in 1896 in Idaho and was called “black measles” [53]. If left untreated, the disease may be severe and often fatal. Currently, successful antibiotic therapy has limited the number of deaths; for example, in the USA, approx. 5–10% of rickettsiosis deaths are caused by RMSF per year [54]. However, in Brazil, Horta et al. [55] showed 20% to 40%, depending on the region. In areas where diagnosis and treatment are difficult, the mortality rate is 80%. Current media data indicate an extremely high incidence of RMSF, including fatalities. Most cases are the result of tick bites [56]. However, Vilges de Oliveira et al. [57] highlighted the possibility of *R. rickettsii* infection from accidental exposure in research laboratories or from percutaneous needle sticks in healthcare settings, which has already been reported in Brazil [57].

In Europe, frequently diagnosed tick-borne diseases include two entities: Tick-Borne Lymphadenitis (TIBOLA) and DEBONEL (*Dermacentor-Borne Necrosis Erythema Lymphadenopaty*). TIBOLA was first described in France in 1997 in a 39-year-old female patient [52,58]. The etiological factor was *R. slovaca*, which was transmitted by the tick, *D. marginatus.* DEBONEL has the same etiological factors and symptoms and was first described in Spain in 2004 [56]. In both cases, a characteristic symptom is a crust located on the scalp in most patients, and painful lymphadenopathy [52,58,59,60]. For these disease entities and for others with similar symptoms and caused by related tick-borne pathogens (of the genera *Francisella* and *Bartonella*), the disease syndrome was called SENLAT (*Scalp Eschar and Nack Lymphadenopathy After Tick*) [61].

Diseases caused by *R. rickettsii* and *R. slovaca* are typical entities of a group of spotted fevers. In contrast, *R. prowazekii* and *R. typhi* are etiological factors that cause classic typhus. These diseases occur with a centrifugal rash distribution. In 1916, Henrique da Rocha Lima first described *R. prowazekii* as the pathogen that causes typhus. It is currently assumed that this disease occurs in people living in crowded places where lice are common [62]. The initial symptoms are nonspecific (muscle pain, headaches, chills, and malaise). After a few days, a rash appears on the body, except for the face, feet, and hands [42]. Interestingly, *R. prowazekii* is the only species of the *Rickettsia* genus that can cause a latent infection manifested by recurrent Brill-Zinsser disease [1,42]. Another TG species, *R. typhi*, is considered the etiological agent of murine typhus (also called epidemic typhus). In case of infection with these pathogens, the characteristic necrotic change on the skin (crust) is not observed [1,63].

The number of rickettsiae infections in certain countries is increasing. The data are often inaccurate. As reported by the Rickettsiosis Subcommittee to the Tick-Borne Disease Working Group in 2018, 5500 cases of spotted fever in the SFG group were reported to the CDC, USA, but the authors indicated that this was not a real number. According to them, one of the main problems is the lack of simple tests for quick and easy diagnosis directly at the site of primary medical care, but also a lack of awareness among physicians regarding the diagnosis and methods of treatment of tick-borne diseases [64,65]. At the same time, the authors of the report noted that treatment with antibiotics is not always effective, as few of them have therapeutic effects.

Much attention has been devoted to the co-infection of hosts with various pathogens. This may result from the infection of one feeding tick with several pathogens or from the feeding of several ticks infected with various pathogens. The latter is most often observed in animals. Breeding and domestic animals that spend a lot of time in open spaces are exposed to numerous attacks from ticks. Infections caused by several pathogens are called tick-borne coinfections (TBCIs). Multiple tick-borne infections in humans and animals have a significant effect on disease diagnosis. In the USA, the most common co-infections include Lyme borreliosis with babesiosis [66,67]. The symptoms are definitely more severe, and the course is longer than in the case of single infections [68]. Nyarko et al. [69] also observed that superinfection with *A. phagocytophilum* produced a more severe form of Lyme boreliosis, e.g., by increasing vascular permeability for spirochaetes. In a literature review prepared by Boyer et al. [70], the authors indicated that TBEV (*Tick-borne encephalitis virus*) was the most common factor of co-infection in Europe. In a two-pathogen system, that included TBEV and *B. burgdorferi* sl. The next double co-infection with regard to incidence was a system involving *A. phagocytophilum*, which was detected in patients with erythema migrans (*Borrelia* spirochaetes). The authors noted that the majority of the reported cases involved symptoms of one of the tick-borne co-infections. They also pointed to a lack of international guidelines allowing for the effective diagnosis, examination, and description of cases of co-infections with pathogens. A common practice in diagnosing tick-borne diseases is to target the most probable infectious agent in a given area, and when it is found in the patient, the search is not continued. This provides incomplete information on possible coinfections with tick-borne pathogens.

Tijsse-Klasen et al. [71], based on the analysis of tick co-infection with *Borrelia afzeli* and/or *B. garini*, as well as *R. helvetica* and/or *R. monacensis* (1.4%), predicted the incidence of double infections with erythema migrans (EM) in Croatia. The examination of skin biopsy specimens confirmed these predictions. In their analysis of pathogens occurring in *Dermacentor reticulatus* ticks in eastern Poland, Zając et al. [72] found double-co-infection amounting for 8.5% of cases; wherein the most common systems were *R. raoultii—TBEV* (4.26%) and *R. raoultii—B. burgdorferii* sensu lato (1.1%). In addition, Raulf et al. [70] showed the coexistence of the genetic material of *Borrelia* and *Rickettsia* at the level of 12.3% for more than 5000 individuals of *I. ricinus.* The authors suggested that the high rate of co-infection with these bacteria results from the fact that they are not competitive with each other. Each species inhabits different niches in the tick; that is, *Rickettsia* live mainly in the salivary glands and ovaries, while bacteria of the genus *Borrelia live* in midgut cells. A meta-analysis conducted by Raulf et al. [73] showed that additive or synergistic interactions occur between *Rickettsia* spp. and *Borrelia* living in *I. ricinus*, resulting in a higher level of co-infection in the vector. Consequently, they increase the incidence of host coinfections. The authors also observed that this kind of co-infection is highly likely to affect the survival of the vector, especially in developing nymphs.

Moutailler et al. [74] found that the co-infection of ticks with several pathogens is not unusual. In a group of 267 female *I. ricinus* harvested from the French Ardennes, 45% revealed the presence of at least two pathogens. Using high-throughput real-time PCR and RT-PCR (for the detection of Tick-Borne Encephalitis Virus—TBEV), the estimated infection rate was 9% for two pathogens, 6.7% for three, 1.9% for four, and 0.75% for as many as five pathogens. Moreover, Moutailler et al. [74] also observed the co-occurrence of symbionts and pathogens in *I. ricinus* females. Although the authors did not observe synergistic or antagonistic associations between these groups, they did not exclude the possibility of symbiont–pathogen interactions.

A literature review conducted by Roche et al. [75] showed that different host species in different regions might have dissimilar systems of pathogen co-infections. Therefore, when looking for mutual correlations, one should consider several variables, such as tick species, developmental stage, sex, or feeding status.

The contemporary human lifestyle facilitates the maintenance of the tick population at a high level, but also a higher overlapping of common habitats. Today, not only forests, suburban meadows, and rural areas pose a threat. There are increasing reports on the direct presence of these arachnids in the vicinity of humans and domestic animals [76,77,78,79]. Therefore, it is crucial to educate medical and veterinary services, but also people, about the potential threat of getting tick-borne diseases. It is also important to develop adequate procedures to prevent these diseases and for effective diagnosis and treatment.

## 6. Metagenomic Analyses and the Future in Research on Tick-Borne Pathogens

Rickettsioses are rapidly spreading in the modern world. This is promoted by climate change, environmental changes made by humans, and the synanthropisation of plants and animals. Therefore, it is extremely important to monitor natural and urban environments. Studying potential vectors and reservoirs for bacteria of the genus *Rickettsia* should be a permanent element in the analysis of contemporary human environments. Currently, methods based on metagenomic analyses provide great support for research on the natural environment, as well as diagnostics. Next-generation sequencing (NGS) or nanopore sequencing (NS) changes the picture of infection in both ticks and facilitates the diagnosis of rickettsial diseases in humans. Recent publications have indicated that the use of modern bioanalysis methods to detect and identify *Rickettsia* spp. is the most effective method to date [3,4]. Polsomboom Nelson et al. [80] found that using nanopore sequencing resulted in higher infection rates with *Rickettsia* species (54.5%) than using classic PCR and Sanger sequencing for the *ompB* and *gltA* genes (47.3%). Of equal importance, researchers demonstrated the presence in *I. ricinus* ticks of DNA from *R. helvetica*, *R. monacensis*, and also *R. asiatica*, which has not yet been described in Europe. This is the first time that this species has been described in Polish and Bulgarian ticks, with 50% and 40% infection rates, respectively. In addition, they detected the genetic material of *R. raoultii* in *D. reticulatus* ticks (12%, in Poland) and *R. massiliae* and *R. tillamookensis* in ticks from *Ripicephalus secundus* and *Haemaphysalis inermis* (single samples). In addition, NS has revealed the presence of endosymbiotic bacteria in ticks [80]. Chaorattanakawee et al. [3] indicated a higher efficiency in the detection of genetic material using NGS for old samples from 2015 to 2016, which were tested using classical methods. In this case, new previously undisclosed pathogen species were detected in the tested material.

In contrast to conventional testing methods, the modern metagenomic approach for analyzing genetic material has many advantages. First, it allows the detection of pathogens without prior targeted knowledge. We can simultaneously detect multiple pathogens, which shortens and realistically reduces the cost of analysis. The results obtained are the basis for broad functional and phylogenetic characterization of the pathogen [81]. The limitations of the widespread use of modern methods include a lack of standardization, high analysis costs, and an insufficient number of highly qualified personnel. However, analyses based on metagenomics will allow monitoring of the environment for the occurrence and spread of tick-borne pathogens.

## 7. Conclusions

The development and improvement of molecular techniques have enabled researchers to gain a deeper knowledge of the surrounding world. This indicates the possibility of detecting bacteria in unexplored environments. New molecular tools, such as NGS or NS, have created the opportunity to research and verify current knowledge about tick-borne pathogens and discover new species.

## Figures and Tables

**Table 1:**
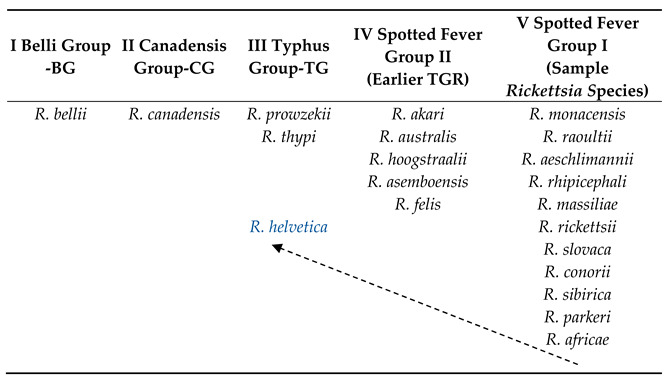
Division of *Rickettsia* species into groups based on phylogenetic analyses (compiled by El Karkouri et al., 2022 [13]).

## Data Availability

No new data were created or analyzed in this study.
